# Theory of Mind and the brain substrates of direct and indirect communicative action understanding

**DOI:** 10.1098/rstb.2023.0497

**Published:** 2025-08-14

**Authors:** Rosario Tomasello, Isabella Boux, Friedemann Pulvermüller

**Affiliations:** ^1^ Brain Language Laboratory, Department of Philosophy and Humanities, WE4, Freie Universität Berlin, 14195 Berlin, Germany; ^2^ Dahlem Center for Linguistics, Freie Universität Berlin, 14195 Berlin, Germany; ^3^ Cluster of Excellence ‘Matters of Activity. Image Space Material’, Humboldt Universität zu Berlin, 10099 Berlin, Germany; ^4^ Biological and Social Psychology, Institute of Psychology, RWTH Aachen University, 52066 Aachen, Germany; ^5^ Einstein Center for Neurosciences, 10117 Berlin, Germany; ^6^ Berlin School of Mind and Brain, Humboldt Universität zu Berlin, 10099 Berlin, Germany

**Keywords:** communicative actions, theory of Mind, brain activation, pragmatics, speech act, language use

## Abstract

Grasping the speaker’s communicative intention based on the verbal utterance and its context is one of the key aspects of human interaction. This ability relies on theory of mind (ToM), that is, the cognitive processing of mental states, beliefs and desires of others. We review evidence showing that brain regions ypically engaged in ToM processing, particularly the medial prefrontal cortex and bilateral temporo-parietal junction, are activated when listeners interpret indirect speech acts. However, other studies show a degree of variability of these ‘ToM area activations’ during indirect communication, while yet others demonstrate that the same areas are activated for specific communicative functions, such as requests, promises or questions *per se*, even when expressed directly. Furthermore, we show that studies on indirectness of communication raise questions about the role of different communicative function types in drawing upon ‘ToM regions’. We attempt to relate cortical area activations to the commitments, assumptions and intentions that the interlocutors commit to when processing specific communicative function types. In essence, all communicative actions, including both direct and indirect ones, have in common that relevant knowledge about others’ intentions and beliefs needs to be processed by the interlocutors.

This article is part of the theme issue ‘At the heart of human communication: new views on the complex relationship between pragmatics and Theory of Mind’.

## 1. Communication as intention recognition

Language serves as a tool for communication, whose main function is to showcase intentions expected to be recognized and acted upon by the addressees. This process involves not only the speaker´s expression of thoughts and desires but also the processing of the conversational partner’s mental states, assumptions and beliefs as they are manifested in their communicative actions throughout the interaction [1,2]. For example, the expression ‘Can you repair this bike?’ can be understood as a *question* (asking whether the recipient is able to repair it) or as a polite *request* to do so. By communicating in a specific context, the speaker conveys specific intentions along with content informative about the speaker’s knowledge, beliefs and assumptions. Thus, recognizing each other’s intentions involves not only identifying *that* the speaker is displaying an intention to communicate but also grasping the specific intention of *what* is being communicated, that is, the action type that the speaker is initiating (e.g. request), along with the specific content thereof (e.g. to repair the bicycle). This process often also requires understanding *how* a speech act has been communicated, whether directly or indirectly. From a linguistic perspective, saying that a speaker A meant something by using a given utterance x ‘is (roughly) equivalent to A intended the utterance of x to produce some effect in an audience by means of the recognition of this intention*’* [1:385]. This perspective highlights the importance of intention recognition and, more generally, recognition of mental states and the (mutual) awareness of these states between communicative partners, often referred to as ‘Theory of Mind' (ToM) [3–7]. In linguistics, the term ‘common ground’ is sometimes used in a similar way, encompassing shared knowledge and assumptions about others’ beliefs, knowledge and intentions [8,9]. Since Grice’s seminal work, the role of ToM in dialogue and pragmatic language processing has been widely explored, with particular attention to its neural substrates. This line of research relates to the growing field of Neuropragmatics, which lies at the intersection of linguistic pragmatic theories and the neurobiology of language [10–17]. In this context, key brain regions found active in tasks requiring ToM processing include the medial prefrontal cortex (mPFC), the left and right temporo-parietal junctions (l/rTPJ) and the precuneus (PCN) (e.g. [18]). However, the extent to which ToM is involved in communication through language remains a subject of ongoing debate [11,19–25]. A key issue is whether ToM abilities are essential for all aspects of pragmatic processing in communication or are specific to certain phenomena (e.g. implicatures, metaphors, irony and various communicative intentions). If the latter, an important question is how these processes are realized in the brain and whether they may vary across different communicative functions.

The present article aims to review previous neurocognitive studies on the processing of communicative intentions, with a particular focus on the involvement of brain regions typically activated during ToM tasks—hereafter referred to as ‘ToM regions’. We begin with a concise linguistic overview of the pragmatic features that characterize different types of communicative actions or ‘speech acts’, followed by a review and discussion of recent neuroimaging studies that elucidate the involvement of ToM regions and other critical cortical regions in speech act processing.

## 2. The universe of speech acts

When we use language to communicate, we employ it not merely to convey information but to express a variety of communicative actions, which are formally described in linguistic pragmatic terms as *speech acts* [26–28]. Each speech act type is characterized by a specific intention: for example, the intention to inform others when making a statement, to obtain something when placing a request or to express one’s valuation/assessment of a fact when complaining or praising. Linguistic pragmatics has offered a thorough theoretical framework for distinguishing these and many other communicative acts [26,28–38], with a set of linguistic-pragmatic features proposed to characterize them differently [2,26,27,39–42], including:

(i) the *communicative function of a speech act and its content,* including the linguistic utterance itself and its role as a tool to perform a specific action, and any accompanying prosodic cues and non-verbal signs [43–47];
(ii) the *communicative setting and other non-communicative actions* in which the utterance is used, such as the environment in which the interaction occurs and the presence of relevant objects;
(iii) the *intentions and assumptions *to which both conversational partners commit during speech act interactions [27,29]. Several of these features are often realized as reciprocal knowledge, forming what is referred to as ‘common ground’ [8,9]; and
(iv) the *action sequence structure* in which a speech act is typically embedded, considering the partner´s actions that can precede and follow a given speech act [2,21,31,33,36,40,48,49].

These pragmatic features illustrate how the mental states of those who partake in conversation need to be mutually represented in their minds, which vary depending on the speech act type. Taking again the example of the sentence ‘Can you repair this bike?’, this utterance may function as either a genuine question or a request, depending on the speaker´s intention, along with content informative regarding the speaker’s knowledge, beliefs and assumptions about their conversational partner (point (iii)). For instance, in the case of a question, the expectation is that the partner can provide an answer, whereas, for a request, the speaker commits to the assumption that their partner is capable of fulfilling it. Such differences may lead to different ToM affordances, potentially activating different cortical brain regions linked to ToM. Furthermore, different speech act types typically elicit distinct responses from the conversational partner (point (iv)), which may additionally contribiute to the understanding of communicative intentions. Consequently, these varying pragmatic features of speech act types may trigger different patterns of brain activation, reflecting diverse linguistic pragmatic and cognitive processes inherent to each speech act type.

Another aspect of social communicative interactions is that the speaker frequently conveys the intended meaning indirectly. A classic example is saying, ‘It is cold here’ as an indirect request for someone to close the window. An indirect speech act, according to classic definitions [29,35] is a speech act (e.g. a *request*) carried out by means of another one (e.g. an assertion). It has been proposed that a costly sequence of inferential steps needs to take place in order for the addressee to recognize the communicative intention of the speaker [35,50]. This inferential chain might involve some implicit knowledge of speech acts, mutually shared background knowledge, some basic reasoning ability and some knowledge about conventions of cooperative conversation [35,50]. Some conventions in conversation are described in the Gricean framework, which includes a principle of general cooperativeness between conversational partners and four conversational maxims [50]. One of these is the ‘maxim of relation’, which emphasizes stating things that are relevant to the conversation, thus guiding interlocutors to contribute information that is relevant and appropriate to the current conversation. In the case where a communication participant states out of the blue that ‘it is cold here’, this maxim appears to be violated, as one may ask whether this remark is relevant and why. A statement about the temperature becomes relevant in the dialogue if it is understood as a request to which the partner can appropriately respond, by closing the window or offering a blanket.

Apart from the additional inferential steps defined as critical to understanding an indirect act [35,50], the essential pragmatic features mentioned above for direct speech acts (see points (i–iv) above) may also play a critical role in shaping the neurocognitive processes of speech acts carried out indirectly. For instance, if two people are at the beach and one of them expresses the intention to go for a swim alone and the other replies, ‘The sea is too rough’, this indirect warning also relies upon the fact that the speaker believes that the sea being rough can be harmful to the addressee plus the attribution of the same belief to the addressed partner. There are diverse motivations for preferring indirect to direct communication. For instance, a further characteristic of such indirect acts is that the speaker may be considered as having made a request when saying 'it's cold here', although they may still deny the intention to have performed the indirect action (‘I just wanted to say that it is cold, but I actually like that’; see [33,51,52]). Also, indirectness may be used to reduce the impact of face-threatening communicative actions as postulated by Politeness Theory [53] (see also section ‘Speech act type, polarity, and politeness’ in [54]).

## 3. The Theory of Mind brain regions in processing communicative intentions

Numerous neuroimaging studies have aimed to identify the neurobiological basis of understanding others' mental states by examining brain activation during tasks such as story listening or reading [55–59], interpreting comic strips [56,60,61], and other related tasks [57,62]. Although these tasks do not directly test different communicative intentions, they have consistently identified brain regions associated with ToM, including the bilateral temporo-parietal junction (r/lTPJ), along with the mPFC and the precuneus (PCN) [18,63–66]. Less frequently, the posterior superior temporal sulcus (pSTS) and the anterior temporal lobe (ATL) (which includes the temporal pole, TP) have also been associated with ToM [67]. Saxe [66] identified the rTPJ and the mPFC as key regions for ToM. She suggests that the rTPJ is crucial for reasoning about others' mental states, such as their beliefs and intentions, while the mPFC is seen as essential for managing shared goals between conversational partners and external objects, enabling joint attention and collaboration [66]. Other studies have argued that the rTPJ and PCN are activated for personal intentions (e.g. grabbing water to quench thirst), while a broader network, including mPFC, bilateral TPJ and PCN, is engaged for social intentions (e.g. buying a gift for a friend) [68,69].

Apart from the role of ToM in understanding the actions of others, extensive research has been dedicated to exploring the role of the ToM brain network in processing the act of communicating with another person. Studies have explored this effect by comparing communicative actions directed towards another person with similar actions performed without the involvement of a communicative partner. For instance, speaking into a microphone to communicate to a person versus simply testing the microphone was associated with activations in the ventromedial and ventrolateral PFC [70]. Furthermore, in a Taboo-like game, describing a target word to an addressee who knew versus did not know it, activated the lateral mPFC, bilateral anterior STS and left TPJ [71]. In contrast, other studies focusing on non-linguistic communicative tasks, where a speaker communicated to their partner a target location on a grid by moving a geometrical shape, found that the right pSTS was active [72,73] and causally relevant [74] for processing the general communicative intention. These findings highlight the activation and function of ToM areas in processing general acts of communication in social interaction, extending beyond tasks focused solely on understanding a story or others' specific verbal actions.

A range of other studies have extended research into the activation of ToM regions in figurative language processing. Understanding ironic sentences, compared with literal ones, has been associated with activation in the bilateral TPJ, mPFC and PCN [75]. Another study showed that ironic versus literal sentence meanings activated a broader network including the mPFC, PCN, STS and precentral gyrus [76] (see [77] for TPJ activation). In the domain of metaphor processing, left [78] and bilateral TPJ activation has been shown to be strongly engaged [79] (see also [80] for the role of TPJ in neurodegenerative disease). Furthermore, a MEG study demonstrated that activation in the bilateral TPJ predicted early post-processing differences between metaphoric and literal sentences, particularly in Broca’s area and the left middle temporal gyrus (MTG) [81]. Furthermore, the TPJ, typically defined as a core region for ToM processing, has also been proposed to function as a cortical hub, integrating attention and memory with higher-level social cognition to support decision-making processes [82].

Although extensive research on the neural basis of ToM in general communication, figurative language and non-communicative tasks has provided results consistent with the role of ToM in such processes, this paper focuses on the role of ToM and associated critical regions in the processing of distinct communicative actions (e.g. request, naming, question, promise etc.). From a theoretical perspective, different speech acts are associated with different intentions and assumptions (as outlined in §2) and thus differ regarding the corresponding mental states of the interlocutors performing or understanding them. We aim to clarify the involvement of ToM-related brain regions and beyond, in processing different speech act types and to investigate whether the activation of these regions varies according to their distinct pragmatic features.

### (a) Direct speech acts

Previous studies have demonstrated that different speech act types are processed very rapidly by the human brain, engaging distinct brain networks [16,83–90]. Specifically, these studies were able to isolate brain activation patterns associated with pragmatic properties or ‘features’ related to specific speech acts by maintaining consistent verbal material, semantic content and visual scenarios, thus ensuring that observed differences were solely due to the function of the utterance in various contexts.

Based on theoretical linguistic consideration, ToM brain regions are likely involved differently depending on the type of speech act, as these acts engage varying assumptions, beliefs and commitments between conversational partners (see §2 for details). Supporting this, a magnetoencephalography (MEG) study [86], which measures magnetic fields generated by neural activity at high temporal and spatial resolution, showed that using the same single word (e.g., flower) for naming or requesting an object, elicited activity in different cortical areas at different timing. Specifically, requests, compared with naming actions, triggered early activation (50−90 ms) in the bilateral motor regions along with the rTPJ. This suggests that communicative actions along with their commitments regarding intentions and beliefs are processed very quickly in speech perception. In addition, the full set of ToM-related regions became activated later (200–300 ms), involving the left and right anterior cingulate, ventral prefrontal cortex (vPFC) and the lTPJ, indicating further processing of speech act and ToM-related information. Further support of differential involvement of ToM-related areas in processing specific speech acts comes from a functional magnetic resonance imaging (fMRI) study [84], which provides a more precise localization of brain activity by mapping blood flow changes to specific tasks or cognitive processes. The same sentence was presented in different contexts, either as a request versus reply (‘You will analyze the survey data this week’) or as a promise versus reply (‘I will analyze the survey data this week’). Requests, compared with replies, activated core ToM regions (mPFC, rTPJ and lTPJ), while promises versus replies showed similar activation but without mPFC involvement. Notably, stronger mPFC and rTPJ activation was observed for promises compared with requests, suggesting greater ToM engagement for promises. No such activation differences were found between the two reply conditions. However, the study found generally stronger motor cortex activation for requests and promises compared with replies (see more about this below), with less pronounced activation in ToM regions. The ToM involvement was primarily detected using multivariate pattern analysis (MVPA) of fMRI data, where enhanced activity in these areas allowed better classification of requests versus replies, promises versus replies, and promises versus requests. The increased activation for requests and promises, compared with simple naming or replies, might rely on their richer commitment structure, tied to the speaker’s intent to obtain an object by request or fulfil a promised action. This is also linked to the expectation that the partner will be able and willing to comply with the request or that the promised action will be completed. In contrast, naming or replies typically just commit the speaker to the truth and relevance of the stated proposition within the conversation. Note also that promise and request are likely to be followed by certain responses of the receiver (disappointment, negative impression of the speaker, reprimanding response) compared with an assertion, which may also affect the neural responses associated with these speech acts. Altogether, these studies provide some indication that ToM engagement may vary depending on the type of speech act, possibly reflecting the assumptions, beliefs and social motives of the conversational partners.

Other fMRI studies have also reported the involvement of the ToM system in processing the speech act type conveyed by speech prosody [91] and sentences [92]. Significant correlations were reported between mPFC and responses to a questionnaire on speaker perspective [91]. Yet, these studies found cortical area activations of the ToM system as well as activation in emotion-related regions (i.e. amygdala) by collapsing different speech acts (criticism, doubt and suggestions) into one condition against ambiguous (morphed) ones [91] or comparing collapsed activation of various speech acts with control sentences [92]. Therefore, it remains unclear whether the activation of the ToM system in these studies is driven by all speech acts included in these studies or rather by a subset of specific speech acts studied. For instance, expressions of doubt might be more socially engaging than suggestions, or the activations in the amygdala might have been driven by the inclusion of socially threatening speech acts such as criticism or doubts. Alternatively, these activations might have reflected general communicative intention.

**Figure 1.**
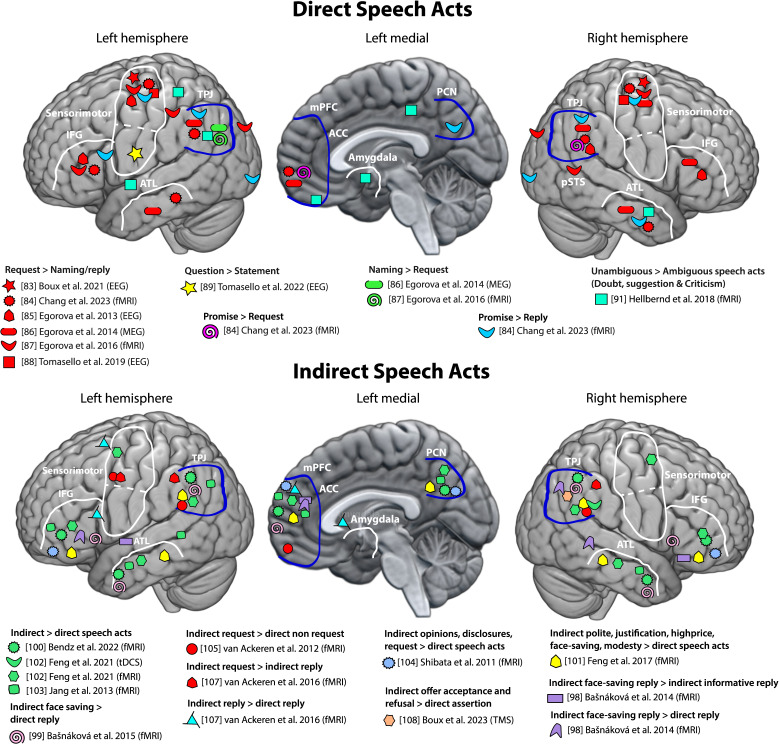
Cortical brain regions involved in direct and indirect speech act understanding and performance. The figure highlights brain regions active in direct (top panel) and indirect (bottom panel) speech acts for the left, left medial and right hemisphere based on evidence from various sources, including electroencephalography (EEG) source analysis, magnetoencephalography (MEG), functional magnetic resonance imaging (fMRI) and transcranial magnetic stimulation (TMS) studies and using a range of different analyses, incorporating univariate contrasts and multi-voxel pattern analyses. Regions circled in blue represent areas identified to belong to the theory of mind (ToM) system [64], while the other labelled regions approximate Brodmann areas or Desikan atlas regions [96]. Note that, here, the locus of peak activation for each study is approximated, as coordinates were not always reported. The dashed line in the sensorimotor region separates the hand motor regions from the face/articulatory motor area. The medial activity shown reflects bilateral generators; however, it was often challenging to assign it to a specific hemisphere. Note that several studies reported activation of the insula, which was not depicted in the figure to avoid clutter. The key regions highlighted include: the temporo-parietal junction (TPJ), which includes or overlaps with the angular gyrus (AG) and the supramarginal gyrus (SMG), though not explicity labeled in the figure; medial prefrontal cortex (mPFC); precuneus (PCN); sensorimotor areas; inferior frontal gyrus (IFG); anterior temporal lobe (ATL); posterior superior temporal sulcus (pSTS); anterior cingulate cortex (ACC); amygdala (top panel adapted from [16], bottom panel created in part from [97]).

In addition to ToM activation, many studies have consistently observed motor cortex activation, which may reflect the action that follows certain speech acts (e.g. grasping and handing over an object after a request). This activation pattern has been consistently observed both in language comprehension and production, with single words or sentences, and in multimodal processing, such as gesture–word combinations [83,86–89] (for review see [15,16]). An electroencephalography (EEG) study, computing source localizations based on scalp-recorded electrical potentials (though with limited spatial accuracy), found that motor cortex activation for requests compared with naming occurs rapidly at around 150 ms after the onset of the speech act [88]. This EEG result confirmed earlier fMRI findings, showing broader involvement of regions, including the bilateral premotor areas (PMC), left inferior frontal gyrus (IFG), right superior temporal sulcus (STS) and anterior inferior parietal sulcus (aIPS) [87]. The motor cortex also appears to be more strongly engaged during commissive (i.e. promising) speech act understanding as compared with simple statements uttered as replies to questions [84]. Furthermore, the same study examined neurological patients with sensorimotor lesions, comparing them with healthy individuals and those with other brain lesions, further confirming sensorimotor cortices having a crucial role in speech act understanding. Specifically, patients showed lower predictability ratings of the addressee’s will in understanding promise actions or of the speaker's will in understanding request intentions as compared with their respective replies (i.e., control conditions) [84]. However, these behavioural results obtained from premotor-cortex-lesioned patients in verbal rating tasks need to be interpreted with great caution, as other work demonstrated a deficit in action word processing in patients with sensorimotor cortex lesions [93]. Pronounced motor cortex activations have also been observed when other speech act types conveyed by speech prosody were processed [89]. In this regard, the study showed that, in spoken language, sentence final pitch (rising for questions, falling for statements) triggers distinct brain responses around 100 ms after critical word onset, where prosodic differences are most pronounced [89]. This timing approximately coincides with the speech act recognition point examined in a gating study as speech unfolds [94]. Notably, question-related activation enhancement was seen within the articulatory sensorimotor cortex, in contrast to the hand motor cortex activations associated with requests [89]. Again, this rapid motor activation may be linked to the expected action of the partner, providing the requested information with the mouth/face, thus highlighting the role of the action sequence following a speech act during understanding. In contrast, naming focuses attention on an object’s features (e.g. colour, shape) rather than partner actions, with studies showing pronounced left angular gyrus (AG) engagement for naming, linked to semantic processing and sensory integration [86,87]. Several of these studies [83,88,89] documented activation differences between speech acts only in the motor cortex, without significant activations in ToM regions. This is likely because of the use of EEG source localization, which has lower spatial resolution and misses deeper activations possibly relevant for intention understanding [95]. Still, a number of studies as described above revealed ToM area activation (i.e. TPJ and mPFC) for speech acts performed directly (see figure 1 (top panel) and electronic supplementary material, table S1).

### (b) Indirect speech acts

Substantial evidence for the involvement of ToM-related regions comes from a range of studies on the comprehension of indirect speech acts [98–107]. Typically, participants are presented with the same linguistic form (a word or sentence) in two different preceding linguistic contexts, one favouring a direct and the other an indirect interpretation. For instance, Bašnáková *et al*. [99] used a simulated job interview scenario where the same sentence *‘*I have read a lot of books in German during college*’*, serves as a direct answer (statement) when replying to the question *‘*How and when did you acquire basic command of German*?’* or as an indirect reply (excuse/justification)*,* in response to *‘*You say that you are fluent in German. Have you followed any certified courses?’. Several fMRI studies using similar designs have consistently shown that ToM-related brain regions, mPFC, bilateral TPJ and PCN, are more active for processing indirect compared with direct speech acts [98–105,107] (see figure 1, bottom panel). These results are typically interpreted as reflecting a greater engagement of the ToM function, as, by assumption, understanding indirect speech acts involves an additional inferential step to grasp the speaker’s intention, which is less immediately accessible than direct speech comprehension [98,99,101]. In addition, other brain regions such as the bilateral IFG and the bilateral MTG, critical regions for language processing, are typically also active for indirect speech act understanding [98,100–104], showing that a widespread network of cortical regions, including language cortical areas beyond ToM, is at work for processing indirectness.

Although all these studies argue for the involvement of ToM brain regions' engagement in indirect speech act understanding, the strategy to compare direct and indirect communicative actions may be related to both ‘what’ is communicated (i.e. the speech act types) and ‘how’ this is done (i.e. (in)directly). Closer examination shows that, for most of the neuroimaging and neuropsychological studies on indirect speech acts published to date, this confound is of potential relevance (see discussion in [54,108]). For instance, in some studies, indirect speech acts served a face-saving function [99,100], or included polite refusal, justifications or praise, or expressed modesty [101] or opinions, disclosures, request refusals [104] and were often contrasted with unspecified direct speech acts (i.e. it was unclear what action the utterance was carrying out). It might well be that part of these activations in the ToM brain regions is related to the type of speech act being carried out indirectly. Other previous studies reporting activations in the ToM system [102,103] do not report any speech act type information about their direct or indirect stimuli, thus making it difficult to interpret the findings reported by these studies related to the aim of the review paper.

Among neuroimaging studies on the processing of indirect speech acts, a few studies have attempted to disentangle speech act type from indirectness, potentially providing insights into the possible interactions between speech act type and indirectness. In a previous study, Bašnáková *et al.* [98] employed two different indirect replies: indirect ‘informative’ replies and indirect ‘face-saving’ replies (excuses and polite refusals)*,* thus addressing the problem of isolating indirectness from speech act types. The analysis comparing these two types of indirect speech acts (face-saving versus informative) revealed increased activation of the anterior cingulate cortex (ACC), including mPFC activation, along with the engagement of other areas, including the rIFG, and areas often associated with emotion processing, such as the anterior insula. The direct versus indirect contrast also showed rTPJ activation and a range of other regions, but this analysis suffers again from the confound of indirectness and speech act type. The stronger involvement of the anterior ACC/mPFC and emotion-related regions for face-saving actions, compared with informative actions, may be due to the richer assumptions about the addressee’s current or future mental state, and/or alternatively, to a deeper and more sustained processing of these states. For instance, these replies functioning as refusals or excuses often carry the implicit concern that the addressee might react negatively.

Another line of studies examined the neural correlates of indirect *requests*, in which indirect requests were realized by a combination of an utterance (e.g. *‘*It is very hot in here*’*) and an image (a closed window), biasing the comprehension of the utterance to indirect *requests* (e.g. to open the window, [105]). These were contrasted with other combinations of sentences and pictures (a desert landscape) resulting in direct speech act understanding, typically as a statement/assertion. Indirect requests versus statements led to activity in the sensorimotor system (bilateral inferior parietal lobe (IPL) and supplementary motor area (SMA)) as identified by a motor task involving finger movements. This aligns with earlier findings described above on direct requests*,* showing consistent motor area activation in processing requests regardless of indirectness (see §2; for a review see [16]). Interestingly, indirect request also showed ToM activation (PCN, mPFC and bilateral TPJs), suggesting the involvement of ToM function processing during comprehension. Nevertheless, as the authors themselves note, their study makes it difficult to tease apart brain activations that were due to the *request* speech act (the 'what' aspect) from those due to the indirectness of the speech act (cf. the 'how' aspect). Hence, in a follow-up study, the authors compared replies that could be interpreted as direct or indirect replies or as a request for action, depending on the questions, in order to tease apart brain activations related to indirectness and speech act type [107]. A contrast between indirect versus direct reply showed significant activations in the mPFC and TPJ, consistent with the common finding that indirect language activates ToM-related regions. However, when contrasting indirect requests versus indirect replies, areas in the sensorimotor system were found to be active (bilateral IPL and left precentral gyrus, i.e. motor regions). Dynamic causal modelling (DCM) connectivity analysis revealed that this activation pattern is most accurately explained by increased effective connectivity from mPFC to the IPL, which have both been proposed to be part of the action-related brain system [109]. This suggests that mPFC-related ToM regions may play a key role in processing indirectness *per se*, whereas the sensorimotor system, particularly the IPL, is more specifically involved in request actions. Thus, a range of different results reviewed here speaks to the issue of ToM regions’ involvement for indirectness or specific speech act types (see figure 1 (bottom panel), and electronic supplementary material, table S2).

A recent study used repetitive transcranial magnetic stimulation (rTMS), a technique that temporarily alters activity in targeted brain areas, to examine the causal role of the rTPJ in understanding indirect speech acts [108]. Sentence stimuli that could be interpreted as either direct or indirect replies, depending on the previous sentence context, were used. In the absence of TMS, indirect speech acts took longer to be processed than direct ones. However, after TMS was applied to the rTPJ, this delay was still detectable when comparing direct statements versus indirect statements (speech-act-matched conditions) but disappeared when comparing direct statements versus indirect offer acceptances and offer refusals (speech-act-unmatched conditions). However, similar to much previous imaging work, this latter ‘unmatched’ condition suffered from the confound that indirectness was varied together with speech act type. Crucially, the TMS manipulation in this study also altered subjects’ performance on a ToM task [110,111], thus providing support that the stimulated region was causal in ToM processing, but only when an additional change of communicative function was involved, not when indirectness was the only differing feature. These TMS results suggest that the rTPJ, a key ToM-related region, is causally involved in processing indirect communicative functions only when additional conditions are met. The fact that TMS affected indirect refusals and acceptances, but not indirect statements, indicates that the causal involvement of this area depends on both factors: indirectness and the type of speech act.

## 4. Concluding remarks and future directions

Recent neuropragmatic research has provided evidence for the involvement of ToM-related brain regions in processing communicative intentions and beliefs of the interlocutors during dialogues, regardless of the type of communicative function under study [71–74,112–114] (for a review, see [69]). However, communicative actions or speech acts are characterized by a range of linguistic pragmatic features, such as the action sequence structure, dialogue structure and assumptions about the mental states (beliefs, intentions etc.) of the interlocutors (see §2). Hence, a broad range of distinct speech act types are intimately linked to processing other people’s assumptions, beliefs and desires, which vary depending on the speech act type. Consequently, the processing of these communicative actions may differently rely on ToM-related and other brain regions, reflecting the specific pragmatic features of each linguistic action.

Examining the neuroimaging literature, we find some support for the involvement of ToM-related brain regions for specific speech act types expressed in a direct manner (figure 1, top panel). In particular, studies have shown that using the same utterance as a tool for requesting compared with naming or replying to a question results in stronger activations of the bilateral TPJ and the mPFC (areas commonly associated with ToM). Similarly, promise actions, as compared with simple replies to a question, also activate similar brain regions more robustly [84]. The increased involvement of these ToM regions may indicate a more complex processing of others' mental states. This includes understanding the speaker’s desire for the requested object and the partner’s willingness to provide it, or the speaker’s intention to fulfil a promised action and the partner’s expectation that the action will be fulfilled. This is not the case for naming or simple reply to actions, for which it is implicated that the stated information is true and/or possibly that the information asserted is new to the interlocutor. Overall, the set of assumptions linked to at least some assertive speech acts appears to be less complex compared with those of requests, commissive, expressive and declarative. In future, it may be useful to investigate naming in more realistic social contexts, where it is used in teaching or testing conversations (see, for example, [83]).

Some researchers suggest that ToM processing is most clearly related to indirect communication. The review of indirect speech acts shows a pattern of ToM area activations similar to that seen during direct speech acts. In both cases, the ‘ToM areas’ mPFC and bilateral TPJ are activated in similar ways, although indirect speech acts consistently show additional PCN activation (figure 1, bottom panel). Methodologically, several studies focusing on indirect speech acts compared indirect with direct actions involving different communicative functions (e.g. indirect request versus direct statement) or provided only limited information about the speech act types realizing directness and directness of communication. This mutual confounding and lack of information make it challenging to determine whether ToM activation in these studies was due to indirectness itself, specific speech act types, or a combination of both [99–105]. A few studies examined the neural correlates underlying indirectness while trying to isolate indirectness from communicative function [98,107,108], a strategy highly recommendable for future studies within the expanding field of neuropragmatics.

The involvement of the mPFC and bilateral TPJ in processing various speech act types is in line with similar evidence on the understanding of figurative languages, such as irony and metaphors [75,76,78,80]. However, a critical question remains: what is the specific role of each of these ToM regions, particularly in speech act processing? Saxe [66] suggests that the TPJ is crucial for reasoning about others' mental states, while the mPFC is involved in managing shared attentional goals between conversational partners [66]. In line with this, one MEG study [86] demonstrated early TPJ activation along with sensorimotor regions during request versus naming comprehension, followed by activation in the mPFC, lTPJ and temporal regions. This seems to suggest that the TPJ may be involved in the early processing of assumptions and intentions inherent to request action, with the mPFC and other regions later integrating this information for shared goals, indicating a reprocessing of ToM-related information. However, it is important to note that the activations reviewed are derived from contrasts between different conditions (e.g. distinct speech act types or direct versus indirect intention processing) applying different tasks, analyses and varying sample sizes, which likely contribute to the variability observed across the studies. Nonetheless, what can be concluded is that certain regions exhibit stronger activation for one condition over another and specifically some regions considered to be relevant for ToM, as identified in various ToM tasks, have been shown to be active for certain speech act types, as well as for the so-called indirect speech acts. However, further work is needed to examine how communicative intention processing can be linked to how these areas work together as a network (see e.g. [107]). Furthermore, the use of independent behavioural measures of ToM is also welcome (see e.g. [100,102,108]), to ensure that the areas found to be active can be interpreted as evidence for the use of ToM function and not of other cognitive functions (e.g. attention, semantic processing, prediction; see [54]), which in some cases are partially linked to the same areas.

Furthermore, other studies point to a distinction between a ‘cognitive’ and an ‘affective’ component of ToM [18,61,64,115], whereas others have emphasized a distinction in neural activation for motivational (desire-related) and epistemic (belief-related) states [116]. In line with the perspective presented here, speech acts may rely on specific parts of the ToM brain system, depending on their distinct affordances. For instance, expressive speech acts might preferentially engage the affective ToM system and the related brain areas, or requests and promises might rely more on the motivational aspects of ToM. Instead, statements and other assertive speech acts that aim at inducing beliefs in the speaker might be processed by parts of the ToM system more sensitive to epistemic states. All of this, in turn, is closely linked to the intrinsic pragmatic features of speech acts, such as the assumptions, commitments and intentions of conversational partners, which vary according to the speech act types. Additionally, the review highlights that, in addition to ToM-related regions, other pragmatic factors contribute to the neural signatures of different speech act types. Notably, the consistent activation of the sensorimotor cortex is observed not only during the processing of direct and indirect requests (relative to naming or statements) but equally for promises compared with assertive acts and for questions as compared with statements [16,117]. This motor cortex activation might reflect the commitment, immanent to these speech acts that an action will follow—a hand-related action in typical ‘give-me’ requests, an action of the actors themselves in the case of promises, and a verbal action in case of a question (see §2). A wealth of pre-existing research clearly shows that complex communicative activities engage multiple cortical regions that seem to be related to action, ToM and social interaction.

A critical concluding remark is that research on the specific engagement of the ToM for specific speech act types is still in the early stages. Future studies should adopt more targeted approaches to clarify the role of each ToM brain region, which can be achieved by bridging together linguistic pragmatic theories, neuroscientific findings, and experimental neurocognitive approaches, an effort that is the ultimate goal of neuropragmatics investigation. Carefully controlled experimental designs should contrast different speech acts, where the only variable is the assumptions and intentions of the communicative partner while holding constant other factors such as the verbal material, the action sequence structure and the mode of communication (whether direct or indirect). This approach would enable researchers to isolate the varying intentions and assumptions associated with different speech act types, allowing a more precise understanding of how ToM and other relevant brain regions contribute to processing various forms of communicative actions.

## Data Availability

Supplementary material is available online [118].
